# High-performance MXene films by sequential bridging

**DOI:** 10.1093/nsr/nwae432

**Published:** 2024-11-28

**Authors:** Xianhu Liu, Jingna Zhang, Chuntai Liu, Changyu Shen

**Affiliations:** State Key Laboratory of Structural Analysis, Optimization and CAE Software for Industrial Equipment, National Engineering Research Center for Advanced Polymer Processing Technology, Zhengzhou University, China; State Key Laboratory of Structural Analysis, Optimization and CAE Software for Industrial Equipment, National Engineering Research Center for Advanced Polymer Processing Technology, Zhengzhou University, China; State Key Laboratory of Structural Analysis, Optimization and CAE Software for Industrial Equipment, National Engineering Research Center for Advanced Polymer Processing Technology, Zhengzhou University, China; State Key Laboratory of Structural Analysis, Optimization and CAE Software for Industrial Equipment, National Engineering Research Center for Advanced Polymer Processing Technology, Zhengzhou University, China

MXenes—a kind of two-dimensional (2D) carbides, nitrides, oxycarbides and carbonitrides of early transition metals—have high mechanical and electrical properties and excellent photothermal conversion, biocompatibility and osteoinductivity [1–4]. The production of high-performance nanocomposites from MXene flakes is of great significance for diverse applications such as flexible electrodes, electromagnetic interference (EMI) shielding and biomedicine [5–7]. Various wet chemical methods, such as layer-by-layer, vacuum filtration, blade coating, wet spinning and superspreading, have been developed to assemble MXene flakes into macroscopic nanocomposites [2,3,6]. Although MXene flakes have been well dispersed in the polymer matrix, the evaporation of solvent during drying would inevitably induce the capillary contraction of MXene flakes, causing their wrinkling and the formation of voids [2,8–10]. The void defects hinder the transfer of stress and electrons between adjacent MXene flakes, significantly degrading the macro-properties of MXene nanocomposites and limiting their practical application. Thus, it is still very challenging to assemble MXene flakes into high-performance nanocomposites.

**Figure 1.**
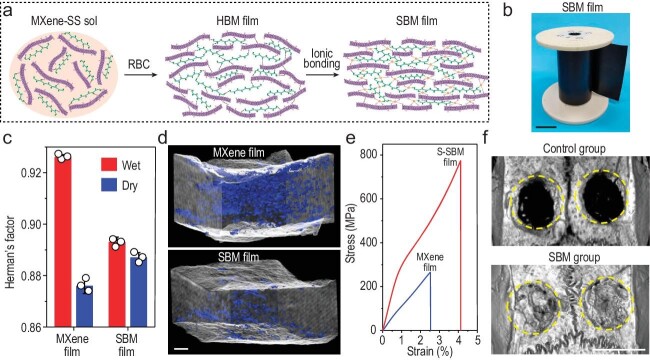
(a) Schematic of the structural evolution in the production process of SBM films. (b) Photograph of a roll of SBM film. Scale bar, 10 cm. (c) Herman's orientation factor of wet and dry MXene and SBM films. (d) 3D void microstructure of MXene and SBM films reconstructed by using nanoscale X-ray computed tomography. Scale bar, 1 μm. (e) Typical tensile stress–strain curves of MXene and SBM films. (f) Micro-computed tomography images of rat calvarial defect areas after bone repair for 8 weeks in control and SBM groups. Scale bar, 5 mm. Reproduced from [11] with permission.

Very recently, Prof. Qunfeng Cheng's group from Beihang University in collaboration with Prof. Xuliang Deng's group from Peking University School and Hospital of Stomatology made an epochal breakthrough in the production of high-performance MXene nanocomposites, which was published in *Nature* [11]. Sequential bridging of hydrogen and ionic bonding in this work was used to restrict the capillary contraction of titanium carbide MXene flakes during drying and freezing of their aligned structure (Fig. 1a), making ultrastrong MXene films with superior osteogenesis. A homogeneous sol consisting of MXene flakes and silk sericin (SS) molecules was first assembled into a hydrogen-bonding-bridged MXene (HBM) film by using roll-to-roll-assisted blade coating (RBC). The HBM films was then immersed in ZnCl_2_ solution to bridge with Zn^2+^, obtaining a sequentially bridged MXene (SBM) film. The production process of SBM films is scalable and a roll of SBM film is shown in Fig. 1b.

After drying, SBM films have a higher retention percentage of alignment than pure MXene films (Fig. 1c), demonstrating that the capillary contraction of MXene flakes is restricted by sequential bridging. Consequently, SBM films show aligned and compact microstructure with a porosity of 4.3% (Fig. 1d), which is much lower than for pure MXene films (16.5%). Because of the aligned and compact structure with improved interlayer connectivity, SBM films provide much higher mechanical properties than pure MXene films (Fig. 1e). The tensile strength (755 MPa) and toughness (17.4 MJ m^−3^) of SBM films are significantly superior to those of previously reported MXene composite films. Additionally, compared with pure MXene films, SBM films have a higher EMI shielding capacity, a higher resistance to sonication damage, repeated mechanical deformation, stress relaxation and oxidation.

A pioneering application of SBM films in bone regeneration was further demonstrated [11]. Because of the good biosafety of MXene flakes and SS molecules, SBM films are highly biocompatible with bone-derived mesenchymal stem cells (BMSCs). When they are implanted into the calvarial defects of rats, SBM films are strong enough to provide a good barrier effect, and MXene flakes and SS molecules can also induce a synergistic effect on the elimination of reactive oxygen/nitrogen species, provoking M2 macrophage polarization and relieving inflammation, and thereby greatly promoting the proliferation and osteogenic differentiation of BMSCs. As a result, plenty of new bone was formed and it filled almost the whole defect area in the SBM group (Fig. 1f). More specifically, the SBM group has a bone tissue volume/total tissue volume (BV/TV) of 77.4% and a bone mineral density of 692 mg cm^−3^. The osteogenesis performance of SBM films is much better than those of commercial guided bone regeneration membranes, establishing the immense potential for application in bone tissue engineering.

In short, Cheng and Deng *et al.* [11] provided a landmark study in the fields of polymer 2D nanocomposites and bone tissue engineering, as it innovatively produced ultrastrong MXene films with superior osteogenesis by sequential bridging. This original work provides a new guideline for producing high-performance nanocomposites from other 1D and 2D nanomaterials by using wet chemical methods. Moreover, this work also pioneers the new applications of MXene films in clinical bone repair and would inspire the design and fabrication of safe, efficient and low-cost bone regeneration materials.
